# The intracellular redox environment modulates the cytotoxic efficacy of single and combination chemotherapy in breast cancer cells using photochemical internalisation

**DOI:** 10.1039/c9ra04430b

**Published:** 2019-08-19

**Authors:** Derick K. Adigbli, Hayley Pye, Jason Seebaluck, Marilena Loizidou, Alexander J. MacRobert

**Affiliations:** Division of Surgery and Interventional Science, University College London London UK d.adigbli@ucl.ac.uk m.loizidou@ucl.ac.uk

## Abstract

*Background*: Photochemical internalisation (PCI) is a light-triggered and site-specific technique that enhances the delivery of therapeutic agents to their intracellular targets using amphiphilic, photosensitizing agents. *Methods*: This study investigated the effect that the intracellular redox environment of 4T1 breast cancer cells exerts on PCI-facilitated delivery of the type I ribosome inactivating protein, saporin, and the topoisomerase inhibitor, mitoxantrone, either individually or in combination. Buthionine sulfoximime (BSO), a clinically used inhibitor of glutathione synthesis, and the singlet oxygen scavenger, l-histidine, were used to enhance the oxidative and reductive state of the cells respectively. *Results*: PCI of saporin at 30 nM was effective in reducing cellular viability, which decreased to 16% compared to “dark” controls (*P* < 0.01). Addition of BSO enhanced PCI efficacy by a further factor of three (*P* < 0.01), but addition of l-histidine completely inhibited cytotoxicity induced by PCI. The combination of the two cytotoxic agents, saporin and mitoxantrone, with PCI, elicited 14% and 17% reduction in cell viability (*P* < 0.01) compared to PCI with saporin alone and mitoxantrone alone respectively. Combination treatment with BSO resulted in a further significant reduction in cell viability by 18% (*P* < 0.01). *Conclusions*: Our findings show the efficacy of PCI can be manipulated and potentiated by modifying the intracellular redox environment.

## Abstract 2

The redox environment modulates photochemical internalization of an entrapped cytotoxic agent. Administration of light depicted by jagged arrow.

## Graphical abstract

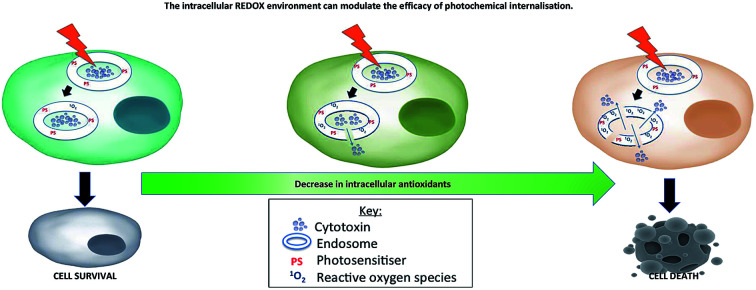

## Background

Drug resistance remains a major cause of treatment failure in cancer chemotherapy. Although many mechanisms contribute to drug resistance, poor accessibility to intracellular targets is a significant factor.^1^ Entrapment and degradation of drugs within endolysosomes is one of the key challenges to overcome for efficient delivery to intracellular targets. This problem applies to macromolecular therapeutics taken up *via* endocytosis as well as smaller agents that are weak bases and susceptible to ion-trapping following protonation within the acidic lysosomes. Photochemical internalisation (PCI) is a novel drug delivery platform derived from photodynamic therapy (PDT) that can enhance the delivery of molecules that become sequestered within endolysosomes into the cytosol.^2^ PCI utilises the basic principles of PDT to facilitate the release of molecules from endolysosomal compartments and requires three components: a photosensitiser, light (of a specific wavelength) and molecular oxygen. Light activation of the photosensitiser results in the formation of reactive oxygen species (ROS), including singlet oxygen (^1^O_2_). Photosensitisers used in PCI are designed to be amphiphilic, and preferentially localise in endolysosomal membranes. The light-induced production of ROS causes localised endolysosomal membrane damage, facilitating the release of contents into the cytosol so that the drug can reach its intended intracellular target and exert its therapeutic effect more efficiently.^1^ The spatial selectivity of the therapy is preserved as “internalised” drugs will be released to act following exposure to light delivered focally to the target site.

PCI has been shown to enhance the *in vitro* and *in vivo* efficacy of many compounds including cytotoxics^3,4^ and immunotoxins^5,6^ in a range of experimental cancer models, as well as gene delivery.^4^ PCI has also been shown to circumvent cellular resistance to treatment using doxorubicin^7^ and even PDT treatment.^8^ A recent *in vitro* study found that PCI also had potential neuronal tissue sparing effects when used to treat a squamous cell carcinoma cell line (PCI30).^9^ Such a desirable safety profile is important for PCI to be considered a suitable addition to the clinical arena. A phase I study, which recruited 22 patients with local, advanced or metastatic solid malignancies reported that disulfonated tetraphenyl chlorin mediated PCI using bleomycin is safe and tolerable.^10,11^

The present study uses a breast cancer cell line, and there is a growing body of preclinical data to support the use of minimally invasive therapeutic options like PDT and PCI in the treatment of breast cancer.^12^ There is significant diversity when assessing treatment response in breast cancer,^13^ underpinned by genetic variations, with four main types based on expression of combinations or absence of ER, PR and HER-2, classified by Perou *et al.*^14^ Current treatments are based on targeting specific genetic variations, such as hormonal agents (tamoxifen and anastrozole) and immunological agents, *e.g.* HER2-blockers (trastuzumab). However, response is often limited by *de novo* or acquired drug resistance.^15^ In metastatic disease, for example, the rate of resistance to trastuzumab monotherapy is 66–88%.^16–18^ Thus, drug resistance remains a major cause of failure in breast cancer chemotherapy. The phase 1 PCI trial included four patients with cutaneous breast metastases which became necrotic following treatment, although surrounding normal skin remained viable, hence our choosing breast as the exemplar cancer in this study.

Two light exposure strategies for PCI with respect to the timing of drug administration have been devised:^19^ (1) “light after” drug delivery – when cells are pre-treated with photosensitiser and drug internalised prior to light application; and (2) “light before” drug delivery – when cells are pre-treated with photosensitiser and light, before the administration of the drug. In this study we assessed how manipulating the reducing capacity of the intracellular environment in breast cancer cells affected the efficiency of PCI using the aforementioned light exposure strategies. The two different approaches used to manipulate the redox environment within a PCI setting were: (i) buthionine sulfoximine (BSO), a synthetic amino acid that irreversibly inhibits gamma-glutamylcysteine synthetase, the enzyme required in the first step of glutathione synthesis;^20^ (ii) l-histidine (LH), a naturally occurring amino acid that has been shown to interfere with redox reactions by scavenging hydroxyl radicals and ^1^O_2_,^21^ and superoxide dismutase. Administration of BSO attenuates intracellular levels of the reducing agent glutathione (GSH), thereby increasing susceptibility to oxidative damage, whereas l-histidine acts to inhibit oxidative damage. In addition by inhibiting GSH production, BSO has been shown to suppress the activity of glutathione peroxidases (GPXs), including GPX4,^22^ which are a group of phospholipid hydroperoxidases that can protect a cell from lipid peroxidation. GPX4 has been shown to play a central role in ferroptosis,^22^ which is a form of non-apoptotic cell death mediated by iron-dependent ROS. The native activity of the GPXs has been shown to be dependent on GSH, which acts as an essential cofactor.^23^ BSO has also been shown to partially reverse drug-resistance in MRP_1_ – overproducing cells associated with decreased levels of GSH and increased intracellular accumulation of daunorubicin.^24^

The study employed a standard PCI photosensitiser, disulfonated *meso*-tetraphenylporphyrin (TPPS_2a_) [4, 7] for PCI of saporin, a cytotoxic 37 kDa type II ribosome inactivating protein, alone and in combination with the topoisomerase inhibitor, mitoxantrone (MTX), a clinically used chemotherapeutic. Although mitoxantrone is a small molecule, it is a weak base and is prone to entrapment in acidic lysosomes. We have previously studied PCI of this agent in multidrug resistant breast and bladder cancer cell lines.^3^ The potential of PCI as a vehicle for the targeted delivery of more than single agent cytotoxins is particularly relevant when considering the translational capacity of PCI to the clinical arena where dual or multiple therapies are commonplace in cancer treatment regimens.

## Experimental

### Cell culture

The 4T1 murine mammary adenocarcinoma cell line stably transfected with the firefly luciferase gene (Luc2) (Caliper Life Sciences, UK) was used throughout. The Luc2 transfection allows bioluminescent imaging; therefore, in addition to *in vitro* investigations it has capacity for future work in *in vivo* pre-clinical models.^25,26^ Cells were maintained in Roswell Park Memorial Institute medium (RPMI-1640) supplemented with 10% (v/v) fetal calf serum (FCS) in a humidified atmosphere of 5% CO_2_/air, 37 °C. Cultures at <90% confluence were routinely trypsinised (1 mg ml^−1^ in 0.2% phosphate-buffered saline (PBS)/EDTA) for propagation. For experimentation, cells were seeded at 10 000 cells/100 μl per well into 96-well plates (Nunc, Roskilde, Denmark).

### Drugs and reagents

The photosensitizer TPPS_2a_ (disulfonated *meso*-tetraphenylporphine, Frontier Scientific Inc.) was dissolved in dimethyl sulfoxide (DMSO) and stored at −40 °C, in the dark, until use. The chemotherapeutics saporin (SAP) and mitoxantrone (MTX) were stored at 4 °C. All other reagents were purchased from Sigma-Aldrich, UK unless otherwise stated. The redox reagents l-histidine (LH), buthionine sulfoximine (BSO) and bovine superoxide dismutase (SOD), were dissolved in PBS and stored at 4 °C.

Cell viability was assessed using the 3-[4,5-dimethylthiazolyl]-2,5-diphenyltetrazolium bromide MTT assay. Absorbance of the reduced derivative formazan at 570 nm, proportional to mitochondrial activity, was used as a measure of cell viability, read on a multiwall plate reader (ELx800 Biotek 4, Bedfordshire, UK).

### Photochemical internalisation (PCI) and photodynamic therapy (PDT)

The terms used throughout are: PDT for TPPS_2a_-alone, without chemotherapeutics and PCI for TPPS_2a_ plus chemotherapeutic agents. The PCI experiments followed either a conventional “light after” or “light before” protocol. In “light after” experiments, plates of cells were incubated for 24 hours under dark (non-illuminated) conditions. At this time medium was removed and replaced with fresh medium containing concentrations of SAP (15 or 30 nM), MTX (0.4 μg ml^−1^) or TPPS_2a_ (0.3 or 0.6 μg ml^−1^), either alone or in combination, for 24 hours. This ensured that in addition to PCI wells, the experiment included a control PDT group exposed to TPPS_2a_, alone. After 24 hours, cells were washed (twice with photosensitizer and cytotoxin free PBS) and received fresh medium. Plates were either illuminated immediately or 4 hours later to investigate the impact of time delay between washing off the drugs and exposure to light. Cells were exposed to a variety of illumination times (60, 90, 120, 150, 180, 240 or 300 seconds), using the Lumisource™ illuminator with peak output at 420 nm (mean fluence 7 mW cm^−1^,^2^ PCI Biotech, Oslo, Norway), so that 120 s at 7 mW cm^−2^ was equivalent to 840 mJ cm^−2^, to activate TPPS_2a_ using its strong blue absorption band. Following illumination, the cells were incubated for a further 24 or 72 hours and viability was assessed using the MTT assay.

The “light before” PCI protocol necessitates incubation and illumination of the photosensitizer prior to exposure to chemotherapeutics. Cells were plated and then incubated under dark conditions for 24 hours. At this time wells destined to receive PCI or PDT were incubated with TPPS_2a_ (0.3 or 0.6 μg ml^−1^) for a further 24 hours. Cells were then washed, media replaced, and plates were exposed to light (as above). Subsequently media was refreshed (for PDT wells, no cytotoxin added) or incubated with varying concentrations of SAP (15 or 30 nM) or MTX (0.4 μg ml^−1^) for PCI-treatment wells. After a 24 hours incubation, the medium was replaced by cytotoxin-free medium incubated for a further 24 hours and viability was assessed using the MTT assay.

All PCI and PDT experiments were carried out under dark conditions and plates wrapped in aluminium foil during incubation to avoid unwanted TPPS_2a_ photoactivation. Cells that were not illuminated or exposed to light are referred to as ‘dark’ controls throughout experimentation. Pilot PDT and PCI studies were initially performed to determine the optimum variable ranges, namely the photosensitizer and light dosing, and the cell washing or ‘chasing’ prior to illumination. The MTT viability assays was applied in these dose-ranging studies at 24 h following exposure to light, and illumination times (light doses) between 60–300 s were investigated.

To determine the effect of washing off the photosensitizer or ‘chasing’, which is well documented in the literature on PCI,^2^ two treatment sequences were investigated. The first (‘immediate’) group comprised cells treated with TPPS_2a_ and SAP and illuminated immediately after drugs were washed off at 24 h. In the second (‘4 hour’) group cells were incubated in media alone for 4 hours after drug was washed off prior to illumination. The ‘immediate’ protocol, for both PDT and PCI, yielded light dose dependent cytotoxicity up to 180 s. In comparison, cells in the ‘4 hour’ protocol group consistently exhibited statistically significant increased cell kill for PCI *versus* PDT: 12% at 60 seconds (*P* < 0.01), 27% at 150 seconds (*P* < 0.01). Therefore, the majority of subsequent experiments were carried out following a 4 hour washout period.

### Combination PCI

“Combined PCI” investigations utilized co-incubation of SAP and MTX at lower concentrations to elicit superior killing than delivery of either chemotherapeutic by PCI alone. “Light before” PCI and “light after” PCI experimental protocols were tested as described above; however, “PCI combo” wells were co-incubated with both 15 nM SAP and 0.4 μg ml^−1^ MTX either before or after illumination depending on protocols. Cell viability was assessed using the MTT assay.

### Redox reagent enhanced or attenuated PCI and PDT

The effect of potentiating or attenuating cellular levels of reactive oxidation species on the efficacy of PCI and PDT was investigated using BSO, or SOD and LH respectively. “Light before”, “light after” and combined PCI protocols were employed as above. However, one of the redox reagents was added throughout drug incubation, illumination and recovery, at the following concentrations: 1.0 μg ml^−1^ BSO, 20, 40 or 80 μM l-histidine and 100 U SOD. Cells were washed twice to ensure removal of residual redox reagents before cell viability was determined using the MTT assay.

### Confocal microscopy

1 × 10^4^ 4T1 (murine) breast cancer cells were seeded in 35 mm diameter glass-bottomed Fluorodishes™ (WPI, UK) and were incubated for 48 hours in full medium (as above) to encourage optimal adherence and sub-confluent cell spreading for imaging. Cells were imaged using an inverted Olympus Fluoview 1000 confocal laser-scanning microscope. Fluorescence confocal images were obtained using a 60 × 1.35 NA oil immersion or a 20 × 0.75 NA objective. ImageJ software (NIH open source) was used to analyze images and obtain mean intensities for delineated regions of interest.

Cells were incubated with MTX, SAP, TPPS_2a_ or a combination thereof for 24 hours and subsequently washed with PBS and incubated in fresh medium for a further 4 hours. At this point, cells were illuminated with blue light (Lumisource™) and imaged after 4 h. Intracellular glutathione expression (in the presence/absence of BSO exposure) was determined by the addition of monochlorobimane (mBCl) which becomes fluorescent when conjugated to low molecular weight thiols including glutathione.^27–29^ The absorption/emission maximum for the glutathione–monochlorobimane conjugate was ∼394/490 nm (Life Technologies, Molecular Probes®, UK). Cells (±BSO incubation) were treated with 40 μM mBCl (in RPMI-1640) for 20 minutes prior to confocal microscopy.^29^

To assess the impact of ROS production on lipid peroxidation, the lipophilic BODIPY® C11 reagent was used as a fluorescence probe (Image-iT® Peroxidation Kit, Life Technologies, Molecular Probes®, UK). The probe was prepared according to the manufacturers guidelines and added at a concentration of 10 μM to treated cells, as described above, 30 minutes prior to confocal imaging. The following combinations of excitation/detection wavelengths were used for fluorescence microscopy: for the reduced form 559/590 nm, for the oxidized form 488/520 nm. Both emission bands are at shorter wavelengths than the porphyrin fluorescence which has peak emission at 660 nm.

### Statistical analysis

Unless otherwise indicated, all experiments were repeated a minimum 4 times. For graphical representations, each experimental value is the average of a 16 well data set. Results are presented as mean values ± standard deviation, and data were analysed using one-way ANOVA with appropriate *post-hoc* analysis, *e.g.*, Tukey's for experiments using combinations of different agents. For images, quantitative data, *i.e.*, those generated from fluorescent intensity evaluation (*e.g.*, ImageJ), were analysed using *T*-tests and/or one-way ANOVA with posthoc analysis, *e.g.*, Tukey's. Significance was set at *P* < 0.05. Specific *P* values are shown within figure legends and/or described in results, as appropriate.

To evaluate whether a synergistic interaction between the two separate therapies applied, we used the following equation to calculate the value of alpha (*α*):
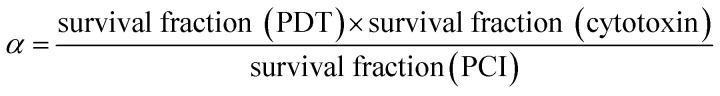


The numerator represents the fractional viability for each separate therapy (*i.e.* PDT using TPPS_2a_ and cytotoxin). The denominator represents the fractional viability observed following the PCI combination treatment. If *α* > 1 then a synergistic effect has been observed whereas an antagonistic effect is denoted by *α* < 1. This analysis has been used previously to identify synergistic effects in PCI.^30^

## Results

The effects of the glutathione synthase inhibitor, buthionine sulfoximine, in combination with PDT or PCI were studied in the 4T1 breast carcinoma cell line to test our hypothesis that treatment with BSO would enhance PCI treatment efficacy. Prior to these studies we sought to demonstrate that treatment of the 4T1 cells with low concentrations of BSO could influence the intracellular levels of glutathione. Since enhanced cytotoxicity using PCI of saporin is triggered by sub-lethal PDT, we also studied the effect of BSO on the PDT response.

### Confocal studies: effect of buthionine sulfoximine on intracellular glutathione expression

**Fig. 1:**
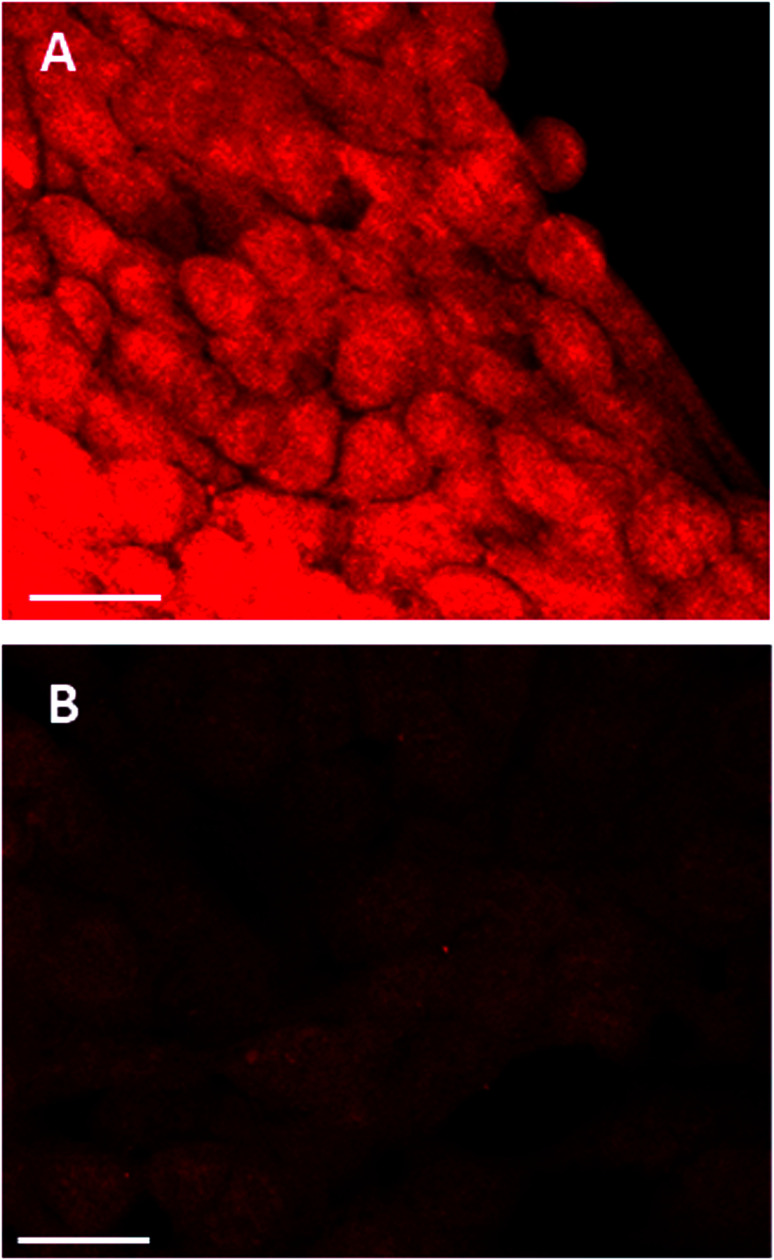
Monochlorobimane–glutathione detection in breast cancer cells. 4T1 cancer cells were treated with mBCl 4 μM for 20 minutes, prior to confocal microscopy either (A) alone or (B) following 24 h incubation with BSO, 1.0 μg ml^−1^; 20× objective, scale bar is 20 microns.

The effect of buthionine sulfoximine (BSO) on the expression of intracellular reduced glutathione (GSH) stores in 4T1 cells was assessed by detection of the GSH fluorescence probe monochlorobimane (mBCl), under confocal microscopy (Fig. 1). Using quantitative image analysis, a four-fold reduction in the mBCl signal in cells treated with BSO compared to controls was observed (*P* < 0.001), which is consistent with a significant drop in intracellular GSH levels and hence alteration of the redox environment.

### Effect of lower glutathione levels on PDT

**Fig. 2:**
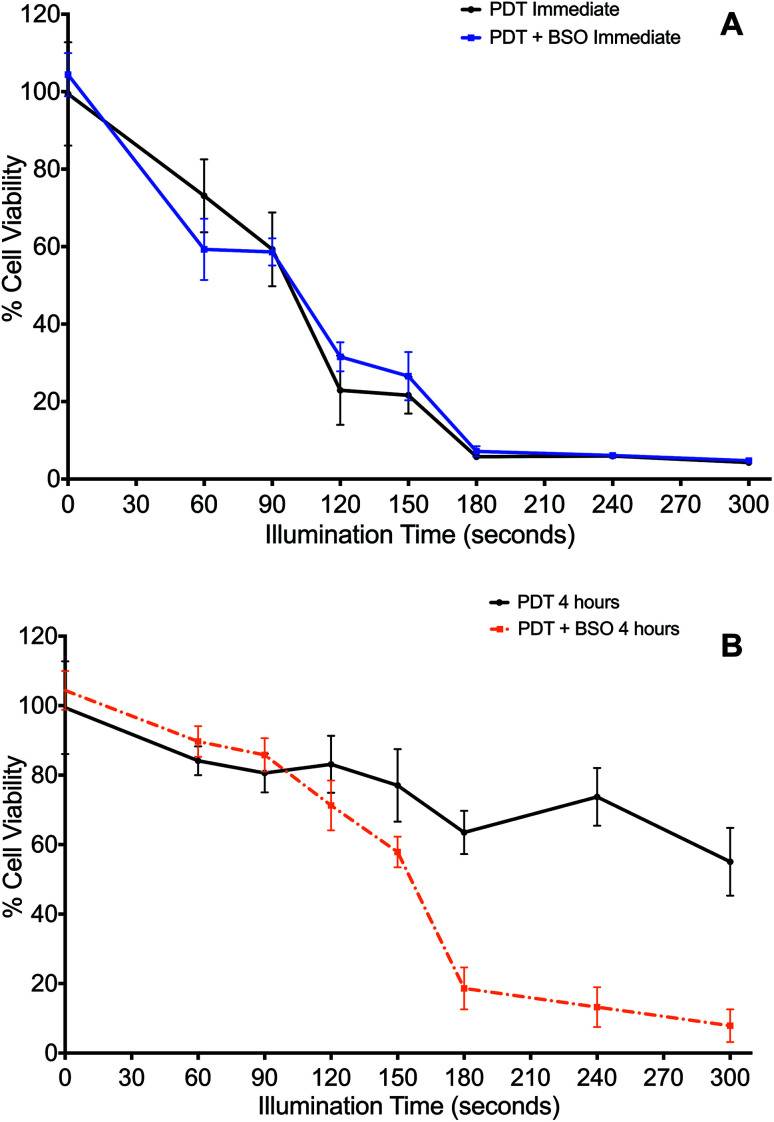
Impact of Buthionine Sulfoximine on TPPS_2a_-mediated Photodynamic Therapy. 4T1 breast cancer cells treated with TPPS_2a_, 0.6 μg ml^−1^, alone. (A) Cells illuminated immediately after TPPS_2a_ washed off, with (blue line) or without (black line) BSO, 1.0 μg ml^−1^; (B) cells illuminated 4 h after TPPS_2a_ washed off, with (orange broken line) or without (black broken line) BSO, 1.0 μg ml^−1^. Cells were illuminated up to 300 s. Cell viability was measured using the MTT assay, 24 h after illumination. Significant cell kill (*P* < 0.01) was shown for all groups >150 s illumination in 2B.

The effect of the glutathione synthase inhibitor, BSO, on PDT was determined when cells were illuminated either immediately (Fig. 2A) or 4 h (Fig. 2B) after washing off the photosensitiser.

Cells treated by PDT utilising the ‘immediate’ protocol, across the range of durations of illumination demonstrated no significant differences between PDT *versus* PDT + BSO (Fig. 2A). In contrast, there was marked difference in cytotoxicity of cells exposed to PDT utilising the ‘4 hour’ washout or chasing protocol, which is the same protocol used for subsequent PCI studies. Viability loss from addition of BSO alone was negligible. Cells exposed to illumination durations ≥150 s exhibited a significantly enhanced increase in cytotoxicity when exposed to BSO + PDT compared to PDT alone. The greatest difference was seen after 240 seconds of illumination, from 74% viability for the PDT group to 13% viability for the PDT + BSO group, which reflects an increase in cell kill of 61% (*P* < 0.01, Fig. 2B).

In the absence of light, the addition of BSO (1.0 μM) caused no significant change in cytotoxicity compared to ‘dark’ PDT alone. However, with illumination, BSO alone killed up to 6% of cells, though given the mechanism of MTT this may represent a reduction in cell metabolism (results for BSO alone not shown).

### Buthionine sulfoximine enhanced PCI

**Fig. 3:**
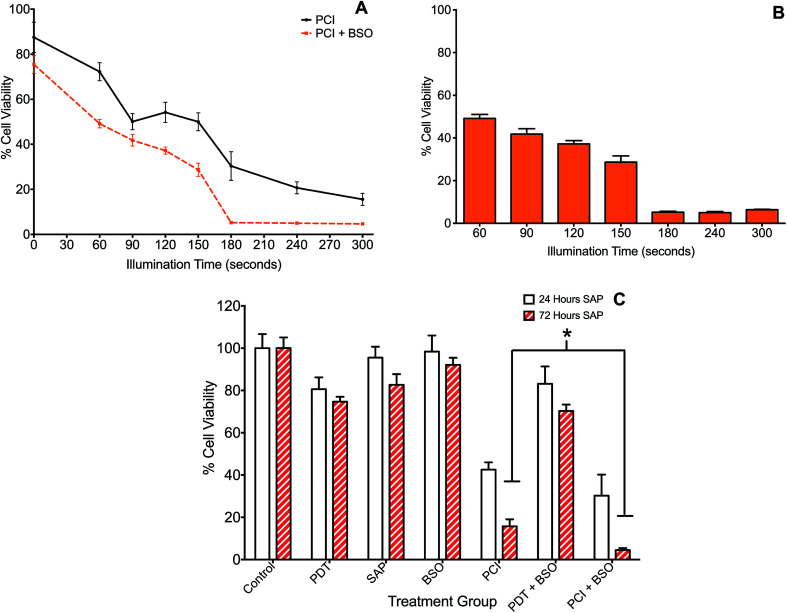
Impact of buthionine sulfoximine (BSO) on TPPS_2a_-mediated photochemical internalisation of saporin. 4T1 breast cancer cells treated with TPPS_2a_ 0.6 μg ml^−1^ + SAP 30 nM (PCI). (A) Cells illuminated for increasing periods up to 300 s with (orange broken line) or without (black broken line) BSO 1.0 μg ml^−1^. MTT assay performed 24 h after illumination; (B) Histogram of PCI + BSO data shown in (A) for comparative purposes. All groups with illumination >150 s demonstrate significant killing (*P* < 0.01); (C) cells were treated with TP or SAP or BSO alone or in combination and illuminated for 120 s and MTT viability assay was performed 24 h (white histogram) or 72 h (red striped) after illumination. Addition of BSO, enhanced SAP-PCI cytotoxicity by 11% at 72 h and a three-fold reduction in viability (*P* < 0.01) resulting in >95% cell kill (*designates *P* < 0.01).

The effect of BSO on PCI (TPPS_2a_ + SAP) was initially investigated with illumination taking place after the 4 h chasing period (the light-after protocol), with the MTT assay carried out at 24 h after illumination. Since PCI is designed to be triggered by sub-lethal PDT, dose ranging pilot studies were carried out to establish the optimum light and photosensitiser doses, with TPPS_2a_ concentrations at 0.3 to 0.6 μg ml^−1^, and illumination times up to 300 s. The dose of saporin used alone (30 nM), had a negligible reduction in viability (<5%) without light. These results showed that illumination times larger than 150 s resulted in significant cell kill (*p* < 0.01 for all) and were therefore unsuitable for our purpose. A combination of 120 s illumination and 0.6 μg ml^−1^ TPPS_2a_ induced a 17% mean reduction in viability (Fig. 2B), which was deemed suitable for subsequent PCI studies. We also carried out measurements at 72 hours post illumination and demonstrated a higher level of cell kill, with generally all groups showing a reduction in viability, corresponding to increased cell death from 24 hours to 72 hours post treatment (not shown). Fig. 3 shows the PCI response with and without addition of BSO for a range of illumination times using the 24 h time-point, and a more detailed comparison at a fixed light dose (120 s) of the data obtained using each time-point. At 72 h after light exposure without addition of BSO, the mean viability of the cells treated with PCI was measured as 16% compared to 43% at 24 hours, corresponding to 2.7-fold higher toxicity (*P* < 0.01). At 72 hours (no BSO), PCI as a treatment was much more effective than either PDT alone (TPPS_2a_) or cytotoxin alone (SAP), by 4.7-fold and 5.2-fold (*P* < 0.01). The corresponding alpha values exceed one (*α* = 1.5 and 3.8 respectively) which is consistent with a synergistic interaction between PDT and saporin.

Addition of BSO to the PCI protocol resulted in the highest cell kill. At 72 hours, the mean viability of the cells treated with PCI + BSO was measured as 5% compared to 30% at 24 hours, which is equivalent to 25% increased cell kill reaching a total of 95% cell kill compared to the control (*P* < 0.01). Comparison of the two treatment regimens (PCI at 72 hours, *versus* PCI + BSO at 72 hours), there is a statistically significant reduction in viability in the latter group (11% difference, *P* < 0.01) corresponding to a three-fold reduction in viability to give >95% viability loss with addition of BSO.

### PCI: combination chemotherapy (saporin and mitoxantrone)

The administration of multiple therapeutic agents is often utilised in clinical oncology. 4T1 cells underwent PCI of a combination of anti-cancer agents, saporin and mitoxantrone, to assess whether this would enhance cell kill compared to PCI of each agent alone. Additionally, the effect on cell viability of the addition of BSO to the treatment protocols was evaluated. Two regimens were compared: (1) standard “light ‘after”’ and (2) “light ‘before”’ chemotherapy PCI. SAP and/or MTX was added to the appropriate treatment groups either preceding (light ‘after’) or following (light ‘before’) illumination.

**Fig. 4:**
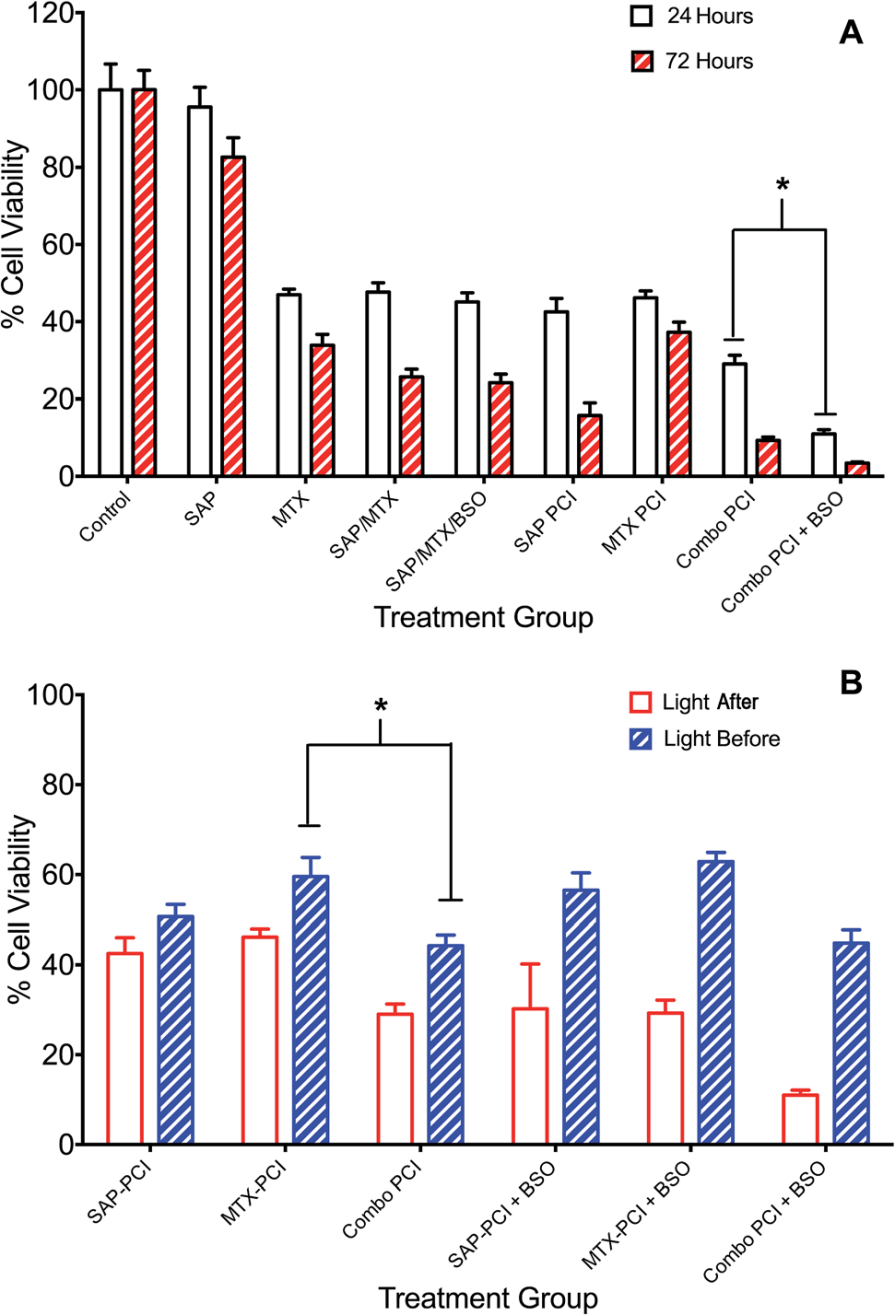
Combination PCI treatment studies. 4T1 breast cancer cells were incubated with different combinations of TPPS_2a_ 0.6 μg ml^−1^, SAP 15 nM, MTX 0.4 μg ml^−1^ and BSO 1.0 μg ml^−1^. To administer PCI, cells were illuminated for 120 s, according to the light “before” or light “after” protocol with cell viability measured using MTT assay 24 h or 72 h after illumination. (A) Cells were treated with photosensitiser or SAP or MTX alone or in various combinations ± BSO and illuminated for 120 s using the light ‘after’ protocol; MTT viability study performed 24 h (white histogram) or 72 h (red striped) after illumination; (B) SAP and/or MTX was added to the appropriate treatment groups either preceding (light ‘after’) or following (light ‘before’) illumination. There were significant statistical differences between all 24 h and 72 h treatments (4A) and all (apart from APS-PCI) light ‘before’ and light ‘after’ groups, which are not designated on the graphs, to avoid cluttering. Also note that the control values for PDT and BSO only with/without light that are shown in Fig. 3 also apply to this figure but are omitted for the sake of clarity (* designates *P* < 0.01).

Overall, combination PCI was more effective than single agent PCI, and more effective than the co-administration of the two agents without PCI (Fig. 4A). This was evident at 24 hours post-illumination, where in the light ‘after’ (LA) group, combination (SAP + MTX)-PCI (Combo-PCI) was associated with 14% and 17% reduction in cell viability (*P* < 0.01, *α* = 0.70 and 1.5) compared to SAP-PCI and MTX-PCI respectively (Fig. 4A). In line with the results presented above, the effect at 72 hours was more pronounced, with a 19% reduction in cell viability observed for Combo-PCI at 72 h compared to 24 h (*P* < 0.01 for both; Fig. 4A). In the light ‘before’ (LB) group, Combo-PCI did not significantly increase cytotoxicity compared to SAP-PCI, although MTX-PCI toxicity was enhanced by 15% (*P* < 0.01, *α* = 1.28; Fig. 4B). Overall, Combo-PCI was more effective with the LA protocol, which produced 15% increased cytotoxicity compared to the LB protocol.

The addition of BSO generally potentiated PCI toxicity for both Combo-PCI and single agent PCI. Addition of BSO to Combo-PCI using the LA protocol had the best and most consistent outcome in terms of toxicity: it resulted in an 18% (*P* < 0.01, *α* = 2.58; Fig. 4A) reduction in cell viability compared to Combo-PCI (24 h). Finally, BSO-enhanced Combo-PCI (LA) produced 34% reduction in viability (*P* < 0.01, *α* = 3.85) compared to the combination of the non-PCI control (*i.e.* all agents BSO/Sap/MTX). Using the LB protocol, addition of BSO to Combo-PCI did not potentiate cytotoxicity, however, it did result in increased cytotoxicity for single agent PCI with a 12% and 18% increase in cytotoxicity respectively (*P* < 0.01, SAP-PCI and MTX-PCI).

### Confocal study: lipid peroxidation

The effect of ROS generation on lipid peroxidation was studied using a lipid peroxidation fluorescence probe (BODIPY® C11), which localizes throughout lipid membranes within a cell. Unsaturated lipids are especially prone to damage by singlet oxygen to form lipid hydroperoxide intermediates. Following oxidation of the probe, there is a shift in peak fluorescence emission from ∼590 nm (red channel) to ∼510 nm (green channel – the oxidized form). As both forms of BODIPY® C11 are spectrally well separated, this property can be used to quantify the fractions of oxidized and non-oxidized probe using a fluorescence ratio assay with confocal microscopy.^31^ A lower red to green intensity ratio therefore corresponds to a higher degree of oxidation and *vice versa*. The fraction of oxidized probe (*i.e.*, the fractional oxidation) is then estimated from the ratio of green intensity/(red intensity + green intensity).

**Fig. 5:**
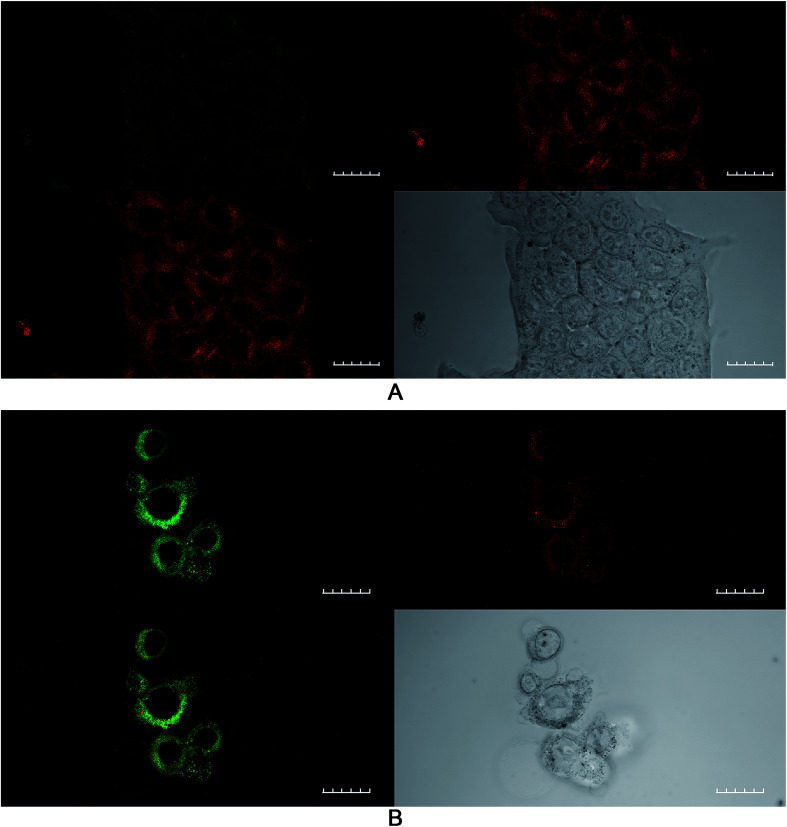
Lipid peroxidation imaging using confocal microscopy after PDT treatment. 4T1 breast cancer cells were incubated with TPPS_2a_ 0.6 μg ml^−1^ alone (PDT) for 24 h before washing and a 4 h incubation period with fresh medium without photosensitizer. (A) Control cells without light exposure; B cells were illuminated for 150 s. The lipid peroxidation probe was added to the cells at 10 μM and incubated at 37 °C for 30 minutes. Top right shows control cells in red channel, top left in green channel, bottom left shows merged channels and bottom right shows white light image. (B) A significant increase (*P* < 0.01) in green fluorescence corresponding to oxidised lipid was observed in the illuminated group compared to ‘dark’ control A. 60× objective, scale bar shown is 20 microns.

**Fig. 6:**
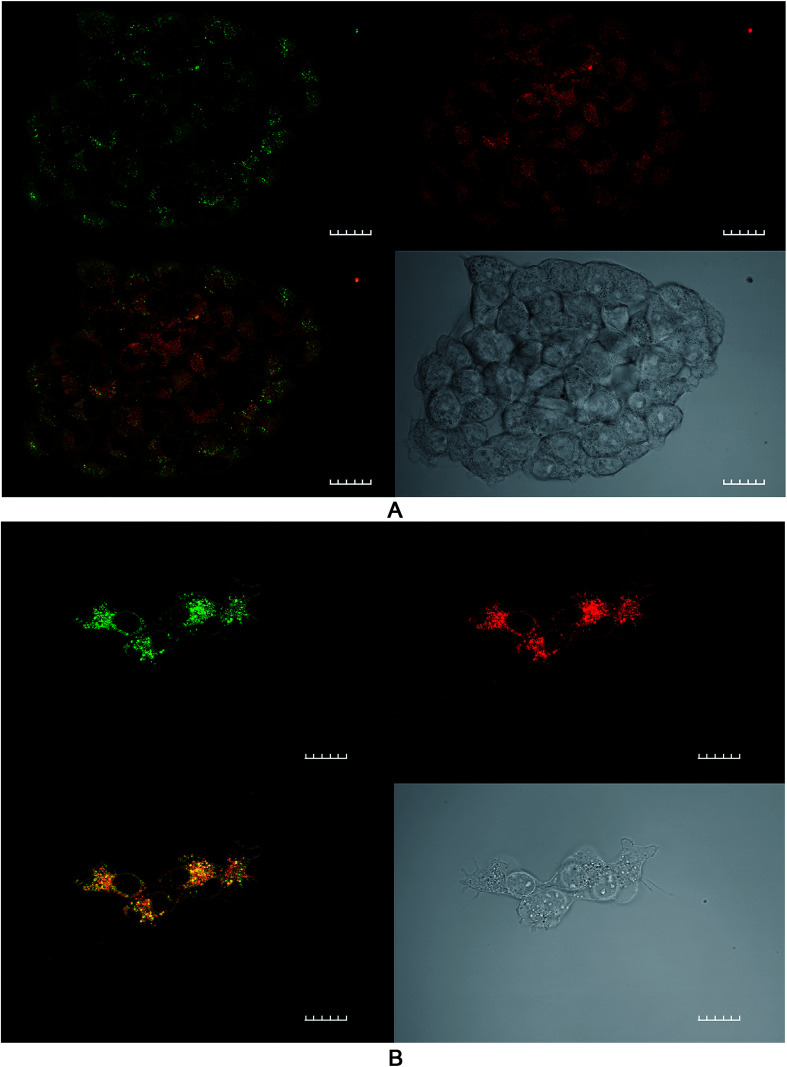
Lipid peroxidation imaging using confocal microscopy after PCI treatment. 4T1 breast cancer cells were incubated with TPPS_2a_ 0.6 μg ml^−1^ + SAP 30 nM + BSO 1.0 μg ml^−1^ for 24 h before washing and a 4 h incubation period with fresh medium without addition of the compounds. Cells in were then illuminated for 150 s and the lipid peroxidation probe was added to the cells at 10 μM and incubated at 37 °C for 30 minutes. Top right shows control cells in red channel, top left in green channel, bottom left shows merged channels (mainly yellow due to combination of red and green colours) and bottom right shows white light image. (A) Cluster of viable cells (B) small cluster of cells with distorted cell membranes and nuclear appearance consistent with cellular distress. A significant increase (*P* < 0.01) in green fluorescence corresponding to oxidised lipid was observed compared to ‘dark’ controls. 60× objective, scale bar shown is 20 microns.

4T1 cells were initially treated with SAP, TPPS_2a_ and BSO either alone or in different combinations ± light (as per 4 h PCI protocol). Fig. 5 illustrates the impact of TPPS_2a_ on the degree of intracellular lipid peroxidation in the 4T1 cells under both ‘dark’ conditions (Fig. 5A) and following 150 seconds of illumination (Fig. 5B). This period of illumination was selected on the basis of the results shown in Fig. 2 and 3 which correspond to a sub-lethal dose relevant to PCI. Ratios of the two emission peaks were compared across the same groups of cells, as shown in Table 1. For control cells under ‘dark’ conditions, the red fluorescence channel signal was 3.4-fold more intense than the green signal, and the green : red channel intensity ratio was 1 : 3.4. Following illumination of 150 seconds, there was a marked diminution of the ratio between red and green signal intensities (Fig. 5B) with comparable intensities instead observed for both channels. The values calculated for the fractional oxidation following illumination increased from 0.23 to 0.53 (Table 1). The combination of BSO + SAP resulted in a green : red channel ratio of 1 : 1.9, corresponding to a fractional oxidation value of 0.34, which is higher than control levels. Addition of SAP alone gave the same result as the control. The effect of PCI [TPPS_2a_ + SAP + BSO + light (150 s)] on the cells is shown in Fig. 6A. A ratio of 1 : 1.9 (green : red channels), equivalent to that seen with BSO + SAP was observed. However, it is important to note that the maximum green signal was 36% greater than that observed with BSO + SAP (*P* < 0.01). Fig. 6B shows a smaller group of 4T1 cells that appear distressed after the same treatment regimen. These cells have a significantly greater intensity of both green and red signals, compared to the other confocal images, and appear to be undergoing the early phases of cell death. The ratio of green : red was 1 : 1.5, thus indicating a significant increase in lipid peroxidation compared to untreated controls.

**Table 1:** Ratio analysis of lipid peroxidation fluorescence probe (BODIPY® C11) for each treatment

Treatment	Ratio of red : green intensities	Fractional oxidation
PDT dark control	3.4	0.23
PDT	0.9	0.53
BSO + SAP	1.9	0.34
PCI + BSO	1.5	0.4

The ratios of the red (non-oxidised form) divided by green (oxidized form) intensities and the fraction of oxidized probe are presented. Values were calculated as described in text from Fig. 5 and 6. The PDT dark control reading is with photosensitizer only, no light.

### Effect of enhanced intracellular reducing capacity induced by ROS scavengers

**Fig. 7:**
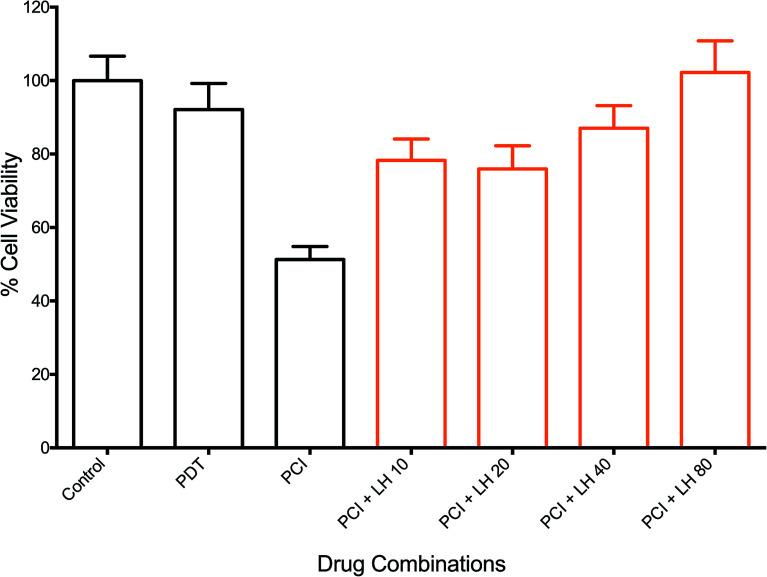
Effect of l-histidine (LH) on photochemical internalisation of saporin. TPPS_2a_ 0.3 μg ml^−1^ + SAP 15 nM + light (90 seconds) ± LH (increasing doses up to 80 μM). All LH groups showed significant reversal of the PCI effect with a maximum of 51% for LH 80 μM (*P* < 0.01; not designated on the graph). 4T1 cell viability measured using MTT assay 72 h after illumination. Controls shown are with TPPS_2a_, but without light.

The effects of ROS scavenging, using l-histidine (LH), on the therapeutic efficacy of PCI were investigated. Fig. 7 shows the response of increasing doses of LH (10–80 μM), incubated 24 h pre- and 24 h post-illumination, on the PCI cytotoxicity. A dose dependent inhibition of PCI-induced cell kill was observed. This ranged from 27% inhibition for LH (10 μM) to 51% for 80 μM (all at *P* < 0.01). The latter completely inhibited PCI induced cytotoxicity with no statistically significant difference from control values. Interestingly no significant reversal effect was seen for PDT + LH even at 80 μM (data not shown) although there is only limited cell kill induced by PDT alone. The effect of SOD was also investigated, using the standard light ‘after’ protocol (TPPS_2a_ 0.6 μg ml^−1^ + SAP 30 nM + light (180 s) ± SOD at 100U). Cells treated by PCI with SOD at 100 U, 24 h pre- and 24 h post-illumination, showed a 43% inhibition (*P* < 0.01) in cytotoxicity, compared to PCI alone. SOD at 100U had no significant effect on PDT-induced cytotoxicity (data not shown).

## Discussion

Photochemical internalisation employs sub-lethal photodynamic therapy to enhance the delivery of bioactive agents that are prone to sequestration in endosomes and lysosomes. In both PDT and PCI, the activation of a photosensitizer leads to the formation of ROS including singlet oxygen (^1^O_2_) superoxide anions (O_2_^−^˙ and OH˙). The cellular phospholipid membrane and organelle membranes are key targets of PDT since they are rich in polyunsaturated fatty acids (PUFA), which readily react with ^1^O_2,_ to generate lipid hydroperoxide intermediates. Superoxide and its protonated form 
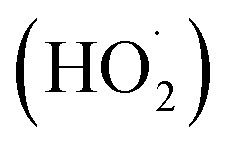
 can also oxidize membrane components directly,^32^ or indirectly *via* its rapid reaction with intracellular nitric oxide to generate the powerful oxidant, peroxynitrite. The subsequent decomposition of the lipid hydroperoxides results in the generation of ROS such as peroxyl radicals. Wagner and colleagues^33^ reported that the sensitivity of PUFAs to oxidative damage increases with the number of double bonds per fatty acid molecules, thus the more unsaturated a fatty acid the greater the reducing power it possesses. Of particular importance when considering the effect of photosensitiser mediated ROS generation in the membranes of organelles is the fact that 30–80% of the mass of the biological cell membrane is made up of lipids. PCI represents a developmental step of PDT, retaining its fundamental properties of light, photosensitizer and oxygen dependent ROS production, but with the effect restricted to subcellular domains,^34–36^ in particular, the phospholipid membranes of endosomes and lysosomes. Localized ROS production within a lipid rich environment facilitates the production of lipid hydroperoxides, particularly when antioxidant capacity is overwhelmed.^37^

By interrogating the relationship between photosensitizer activation in PCI and the intracellular redox environment of a cell, we investigated whether it would be possible to form a greater understanding of the influence of reactive oxygen species (ROS) production on PCI and optimize its efficacy. The main aim of this study was to investigate the effect that the intracellular redox environment exerts on the efficacy of PCI-facilitated drug delivery of SAP and/or MTX. The experiments performed were designed to initially determine whether or not TPPS_2a_ based PCI could be used to enhance the delivery and consequently the cytotoxic efficacy of two cytotoxic agents (SAP and MTX). The balance between oxidation and reduction is vital to the normal functioning of a cell and abnormal redox states have been shown to confer resistance to cytotoxic agents in cancer chemotherapy.^38^ The reducing capacity of a cell is determined by the expression of reducing agents/enzymes including glutathione (GSH), glutathione peroxidase (GSHP_*x*_), catalase and superoxide dismutase (SOD), relative to the oxidative load. These reducing agents act to prevent the toxic and mutagenic effects of ROS, for example, SOD catalyzes the conversion of the reactive O_2_^−^˙ to H_2_O_2_ + O_2_, the former being subsequently detoxified by catalase.^39^ Buthionine sulfoximine (BSO) provided a suitable attenuator of the reducing capacity of the cell, as discussed further below, and has been previously used in combination with PDT.^40^ Saporin (SAP) was the main cytotoxin utilized since it is an ideal agent for investigating PCI owing to its poor native uptake into the cell coupled with its high toxicity once internalized and liberated into the cytosol.^5,41–46^ The choice of TPPS_2a_ as the photosensitizer, which has been widely used in previous PCI studies as a prototype PCI photosensitizer, is also attractive since it is not a substrate for the ABCG2 efflux pumps that are responsible for chemotherapy multidrug resistance.^47^

In the initial part of this work, the feasibility of using PCI for treating 4T1 breast cancer cells was examined. In order for PCI to function optimally, the PDT effect should be sub-lethal, therefore preliminary studies were conducted to optimize the treatment dosimetry. Pre-treatment of cells with photosensitizer plus chemotherapeutic (SAP or MTX) prior to light exposure is the basis of the ‘light-after’ protocol that was mainly employed in this study. This enables the co-localization of both TPPS_2a_^1,48,49^ and chemotherapeutic agent within endosomes. As shown in Fig. 3, we demonstrated that PCI could significantly enhance the cytotoxicity of saporin, in agreement with other PCI studies.^4^ We then sought to investigate whether addition of BSO could further enhance the efficacy of PCI, following preliminary studies of the effect of BSO addition on PDT, based on the hypothesis that PCI efficacy can be manipulated by modulating the intracellular redox environment. BSO is an irreversible inhibitor of γ-glutamylcysteine synthase that causes depletion of GSH in tumours and normal tissues. Thanislass and colleagues^50^ demonstrated that sustained treatment (several days) using BSO in male rats resulted in chronic suppression of GSH levels, which was associated with an overall reduction in antioxidant defenses owing to diminishing activity of catalase, superoxide dismutase, and glutathione peroxidase among other scavengers of ROS. In this study, monochlorobimane (mBCl) was chosen as a fluorescent probe (Fig. 1) for intracellular glutathione levels. The 4-fold reduction in the mBCl signal for 4T1 cells treated with BSO under the same conditions as used for the PDT/PCI studies *versus* untreated cells supports previous findings^51^ where there was significant suppression of GSH and GSSG levels in 4T1 cells after 48 h incubation with 5 μM BSO. Supporting evidence for the role of BSO in enhancing the oxidative capacity of a cell is seen in the lipid peroxidation studies using a lipophilic C11 fluorescence probe (Fig. 5, 6 and Table 1). The C11 fluorescence probe will however be present in other membranes apart from the endolysosomes, and it was also added after light treatment in order to avoid PDT-induced damage by singlet oxygen attack or photodegradation by the light source. Although lipid hydroperoxides are relatively long-lived, it has recently been proposed^52^ that they can be degraded to lipid aldehydes *via* radical attack and in the case of PDT by direct interaction between the hydroperoxides and photoexcited sensitiser. This study also confirmed the close correlation between photosensitised membrane permeabilisation and formation of lipid hydroperoxides.

BSO had minimal effect on PDT using the ‘immediate’ illumination protocol (Fig. 2A). However, following the 4 h chasing period, BSO significantly enhanced PDT for illumination durations >150 seconds (Fig. 2B), and an incremental increase in the effect of BSO on PDT was observed. During the 4 h chasing period, dilution of the photosensitizer that is originally located in the cell membrane takes place after its dispersal into the medium surrounding the cells. Therefore, the majority of the remaining photosensitizer after the chasing period is associated with endolysosomal membranes within the cell. The significant potentiation of photocytotoxicity by BSO at this concentration therefore suggests that its oxidative enhancement effect promotes lipid peroxidation within photosensitized endolysosomal membranes. This effect may be further enhanced by the suppressive effect of BSO on GPX4, rendering the cell more prone to the generation of lipid hydroperoxide.^53^ Consequently, BSO + PCI may enhance cell death *via* both apoptosis and ferroptosis.

Co-incubation of BSO with the cytotoxin resulted in significantly enhanced PCI efficacy in a synergistic manner. Comparison of Fig. 3A with Fig. 2B, shows that PCI resulted in progressively higher cell kill than PDT for illumination times up to 300 s. Addition of BSO enhanced the efficacy of PCI for illumination times ≤150 seconds. At longer times the PDT effect in the presence of BSO is no longer sub-lethal therefore the efficacy enhancement of PCI over PDT is difficult to estimate since the MTT assay can be insensitive at low cell viability values.^54^

As shown in Fig. 3C, the effect of prolonging the post treatment duration from 24 h to 72 h was significant and resulted in increased PCI cytotoxicity for both single agent and combination PCI ± BSO. The increased cell kill could represent multiple mechanisms including prolonged time for saporin induced killing. Saporin induces cell kill *via* apoptosis which is a slower process than necrosis, therefore the lower viability measured at the longer assay time after illumination would be consistent with apoptotic cell death. We have previously observed improved cell kill at 96 h *vs.* 24 h using PCI in prostate cancer cells lines using the same photosensitiser.^55^ The effect of BSO is consistent with a mechanistic role for GSH in regulating the rate of production and survival of ROS generated following the activation of a photosensitizer. Consequently, GSH levels within a cell are likely to have a significant impact on the efficacy of both PDT and PCI. However, since PCI requires only a sub-lethal PDT dose, the enhanced therapeutic efficacy induced by BSO will likely be greater for PCI than PDT which relies solely on ROS generation. Furthermore, Berg and colleagues have demonstrated that photo-induced rupture of endolysosomes using PDT is a relatively inefficient means of cell killing, thus the effect of BSO on PCI may be further enhanced compared to PDT.^56^ Manipulation of GSH may therefore be an important consideration when developing PCI protocols. The ability of a cell to enhance its reducing capabilities has been implicated in the development of drug resistance. Multi-drug resistant MCF-7/ADR breast cancer cells were shown to be 30-65-fold more resistant to doxorubicin than wild type and this was associated with 23-fold elevated glutathione-*S*-transferase (GST) activity within the cytoplasm.^57^ Additionally, levels of nuclear-targeted GST were not only elevated in doxorubicin-resistant cancer cells but dynamically rose in response to treatment; the group concluded that GST protects DNA from anticancer drugs.^58^ This suggests that the use of BSO in harness with PCI for counteracting multidrug resistance could also be an interesting concept to explore further.

We also investigated the effect of antioxidants on PCI, as shown in Fig. 7. Cells were treated with the amino acid l-histidine (LH), which is known to scavenge singlet oxygen.^21^ Since LH is a small molecule it should readily penetrate phospholipid membranes and scavenge ROS generated within the membranes. The dose-dependent reversal of PCI indicates that the mechanism is in part consistent with the involvement of the type II process with the photosensitiser-mediated generation of ^1^O_2_, which degrades the endolysosomal phospholipid membranes. We also carried out studies using SOD, which is a much larger molecule than LH, thus intramembrane and intracellular effects are likely not to be significant. Nevertheless, treatment with SOD elicited significant inhibition of the PCI effect whereas no inhibition was found for PDT only. Further studies with smaller SOD mimetics may be useful as previously done for PDT.^59,60^ The findings of the ROS scavenger experiments add further weight to the importance of ROS generation and survival in PDT and PCI. In the case of LH, it is important to consider that a non-enzymatic anti-oxidant can potentially enhance ROS production, and this was observed at higher doses.

Drug combination experiments were performed (Fig. 4) to model better the reality in clinical practice when cytotoxins are rarely used in isolation. We found that combination PCI can be significantly more effective than single-cytotoxin based PCI using the ‘light ‘after’ (LA) protocol, where the combination of both agents resulted in a substantial increase in cytotoxicity, compared to SAP-PCI or MTX-PCI alone ±BSO. The addition of BSO to combination PCI resulted in 89% cell kill. In contrast, the use of the light ‘before’ (LB) protocol elicited only a weak enhancement in cytotoxicity. The mechanism of the LB protocol is unclear but presumes that photooxidative damage to existing endosomes prior to drug loading can enhance cytosolic delivery. This could occur *via* fusing of partially damaged yet still intact endosomes with new endosomes containing the agents that form after light exposure. These fused endosomes may then become unstable and release the agents into the cytosol. It is possible that a larger light and/or BSO drug dose is required for this protocol to be more effective than we observed.

Combination PCI is an area that certainly requires further evaluation in order to establish its relevance to clinical practice in cancer therapeutics. This *in vitro* study has shown that PCI can be used to facilitate the internalisation of two structurally and mechanistically different cytotoxins in unison and that this is better than single-agent PCI alone.

## Conclusions

PCI is an emerging technology that is already demonstrating its translational potential in clinical trials for the treatment of solid cancers. The aim of this study was to investigate the influence of intracellular oxidation and reduction repertoire on the efficacy of TPPS_2a_ mediated PCI of saporin and mitoxantrone administered either as single agents or co-administered for combined therapy. This study provides new insight into the importance of the redox environment of a cell for PCI and offers an effective means for optimizing drug delivery using this platform. Overall the findings are consistent with the hypothesis that the amount of ROS required to liberate endocytosed drugs from their cytosolic vesicles in PCI is significantly less than that required for PDT-induced cell killing. Consequently, a relatively small increase in the ROS-scavenging capacity of a cell may be sufficient to inhibit the low ROS-burden for PCI but have relatively little effect on PDT. It would be interesting to determine whether BSO can improve the selectivity of tumour damage induced by PCI since tumours can exhibit enhanced levels of GSH compared to normal tissue. The combination of cytotoxins for PCI may further widen the therapeutic indices of the chosen chemotherapeutic agents to enable lower dosages and further reduce unwanted side-effects.

## Author contributions

Conceptualization, D. A., M. L. and A. M.; methodology, D. A. and H. P.; software, D. A. and A. M.; validation, M. L. and A. M.; formal analysis, D. A.; investigation, D. A. and H. P.; resources, M. L. and A. M.; data curation, D. A. and J. S.; writing—original draft preparation, D. A. and J. S.; writing—review and editing, M. L. and A. M.; visualization, D. A. H. P.; supervision, M. L. and A. M.; project administration, A. M.; funding acquisition, D. A., A. M.

## Ethics approval

Not applicable.

## Funding

This work was funded by the award of a Grand Challenge research studentship from University College London to Derick Adigbli.

## Conflicts of interest

The authors declare no conflict of interest.

## List of abbreviations

BSO: Buthionine sulfoximine
LH: l-Histidine
LYG: Lysotracker® Green
mBCl: Monochlorobimane
MDR: Multidrug resistance
MTT: 3-[4,5-Dimethylthiazolyl]-2,5-diphenyltetrazolium bromide
MTX: Mitoxantrone
PCI: Photochemical internalisation
PDT: Photodynamic therapy
ROS: Reactive oxygen species
SAP: Saporin
TPPS_2a_: Disulfonated *meso*-tetraphenylporphyrin

## Supplementary Material
